# Insights into the critical role of the PXR in preventing carcinogenesis and chemotherapeutic drug resistance

**DOI:** 10.7150/ijbs.68724

**Published:** 2022-01-01

**Authors:** Xiaxia Niu, Ting Wu, Gege Li, Xinsheng Gu, Yanan Tian, Hongmei Cui

**Affiliations:** 1 Institute of Toxicology, School of Public Health, Lanzhou University, 730000, Lanzhou, China.; 2 Department of Pharmacology, College of Basic Medical Sciences, Hubei University of Medicine, Shiyan 442000, Hubei, China.; 3 Department of Veterinary Physiology and Pharmacology, Texas A&M University, USA.

**Keywords:** Pregnane nuclear receptor (PXR), post-translational modifications, protein-protein interactions, chemotherapeutic drug resistance

## Abstract

Pregnane x receptor (PXR) as a nuclear receptor is well-established in drug metabolism, however, it has pleiotropic functions in regulating inflammatory responses, glucose metabolism, and protects normal cells against carcinogenesis. Most studies focus on its transcriptional regulation, however, PXR can regulate gene expression at the translational level. Emerging evidences have shown that PXR has a broad protein-protein interaction network, by which is implicated in the cross signaling pathways. Furthermore, the interactions between PXR and some critical proteins (e.g., p53, Tip60, p300/CBP-associated factor) in DNA damage pathway highlight its potential roles in this field. A thorough understanding of how PXR maintains genome stability and prevents carcinogenesis will help clinical diagnosis and finally benefit patients. Meanwhile, due to the regulation of CYP450 enzymes CYP3A4 and multidrug resistance protein 1 (MDR1), PXR contributes to chemotherapeutic drug resistance. It is worthy of note that the co-factor of PXR such as RXRα, also has contributions to this process, which makes the PXR-mediated drug resistance more complicated. Although single nucleotide polymorphisms (SNPs) vary between individuals, the amino acid substitution on exon of PXR finally affects PXR transcriptional activity. In this review, we have summarized the updated mechanisms that PXR protects the human body against carcinogenesis, and major contributions of PXR with its co-factors have made on multidrug resistance. Furthermore, we have also reviewed the current promising antagonist and their clinic applications in reversing chemoresistance. We believe our review will bring insight into PXR-targeted cancer therapy, enlighten the future study direction, and provide substantial evidence for the clinic in future.

## Introduction

**Figure 1:**
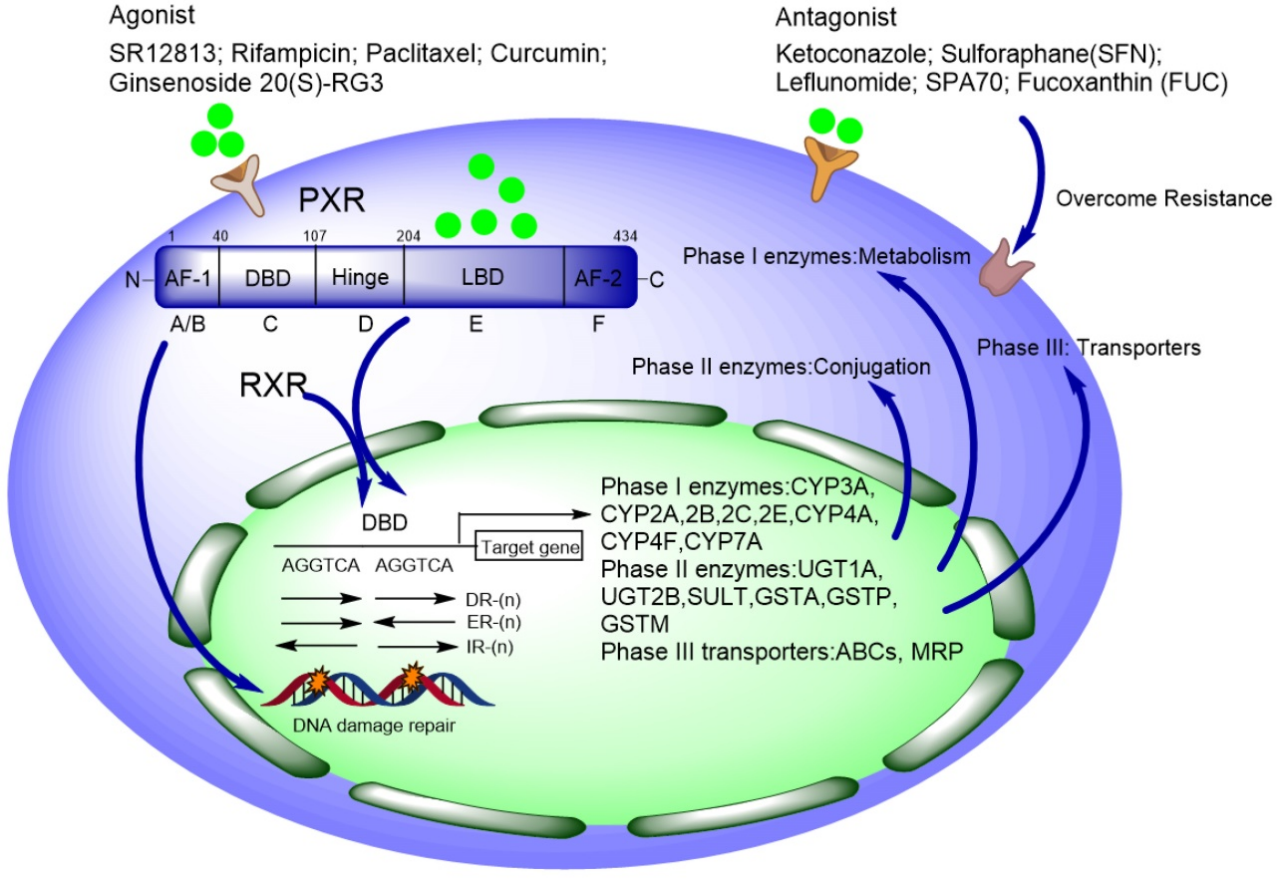
Schematic of PXR' functional domains contain DNA binding domain, hinge region, and ligand binding domain. Xenobiotics (including agonist and antagonist) bind to the ligand binding domain, and then PXR forms a heterodimer with RXRα and translocate to the nucleus, recognizing the consensus sequence on the promoter region of target genes to initiate transcriptional regulation of CYP450 phase I, II, III enzymes. Antagonists can be used to overcome chemoresistance in lab while applied in clinic with caution.

Pregnane x receptor (PXR, SXR, NR1I2) is one of the important nuclear receptors (NRs), which is expressed mainly in the liver, intestine, and colon. Kliewer et al. discovered the full-length cDNA of mouse PXR in 1997, and human PXR (hPXR) was cloned simultaneously 1. PXR plays important roles in disposing of endogenous and xenobiotic compounds and maintains metabolizing homeostasis, therefore, PXR protects the liver against toxic insult from both endogenous and xenobiotic compounds 2, 3. Once xenobiotics as ligand bind to PXR, PXR forms a heterodimer with RXRα and then translocate to nucleus, recognizing the conserved sequence on the promoter region of downstream genes and triggering transcriptional regulation. The PXR responsive elements on the promoter region of target genes (such as CYP3A4 and CYP2B6) are some of the short repeats (AGGTCA), including direct repeats (DR) DR-3, DR-4, DR-5, and the eversion repeats (ER) ER-6 and ER-8 4-6** (Figure 1)**. It is estimated that CYP3A4 is responsible for the metabolism of over 50% of drugs in use today, many of which are either metabolically activated and/or detoxified through this enzyme 2.

Normally, DNA methylation on the PXR promoter is much higher in some cell lines (like Caco-2, HT29, HCT116, and SW48), resulting in silenced PXR expression and finally transcriptionally repressed downstream target genes 7. Besides, the transcription activity of PXR is also affected by some auxiliary co-activators and co-repressors, which have been reviewed in 8, 9. Agonists of PXR include rifampicin (RIF), SR12813, paclitaxel (PTX), et al. 10-12. The ligand-bound activity is thought to make conformational changes to activate PXR by disassociation of co-repressors, and simultaneously recruitment of co-activators 8, 9, 13, 14. These co-factors comprehensively determine how PXR is activated, thereby affecting PXR-regulated response to xenobiotics 14.

**Figure 2:**
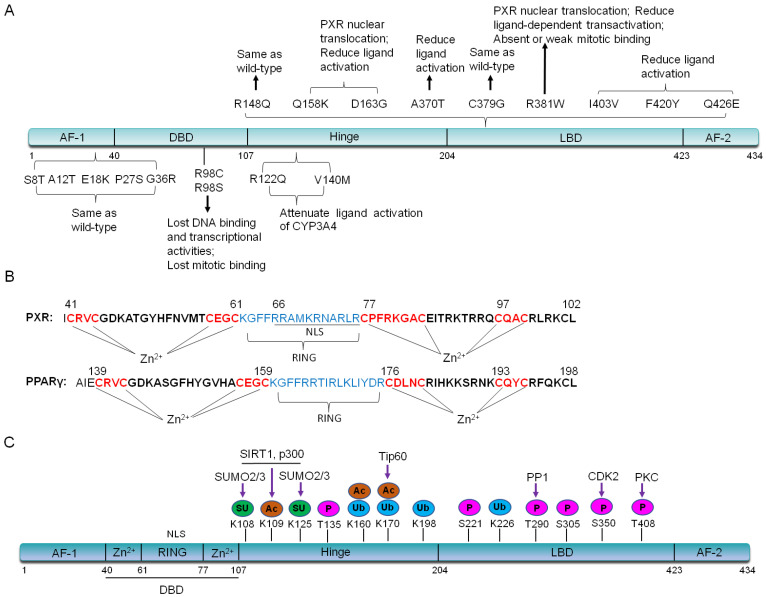
** (A).** Important PXR SNPs on exons and their functions. **(B).** The DBD region of PXR contains a bidirectional nuclear localization sequence (NLS) and zinc finger I (CX2CX13CX2C) and zinc finger II (CX6CX9CX2C) in DBD domain, which is quite similar with that of PPARγ. In contrast, PPARγ has zinc finger I (CX2CX13CX2C) and zinc finger II (CX3CX9CX2C). The RING domain of PPARγ plays E3 ligase activity to degrade NF-κB. **(C).** PXR protein structure can be altered by post-translational modifications. P: Phosphorylation; SU: SUMOylation; Ac: Acetylation; Ub: Ubiquitination.

De mattia et al. have summarized important single nucleotide polymorphisms (SNPs) variants on PXR exons 15-18. In the activating function (AF) -1 and DNA binding domain (DBD), SNPs such as G23C (S8T), G34A (A12T), G52A (E18K), C79T (P27S), G106A (G36R) and C292T (R98C), have no effects on PXR localization whereas affect its ligand-bound capability. C79T (P27S) occurs on exon 2, which is population specific in African Americans with a frequency ranging from 15-20%. This allele has no significant effect on the hepatic CYP3A4 expression 16, 19. C292T (R98C) makes PXR can not bind to promoter region, thus will lose the transcriptional regulation on downstream target genes 17, 20. In the PXR hinge region, the variant G365A (R122Q) and G418A (V140M), potentially decrease affinity of the PXR-DNA binding and attenuate ligand (Such as RIF and corticosterone) activation of the CYP3A4 reporter plasmid in an *in vitro* transient transfection assay 15, 16. It is worthy of note that nine SNPs variants of PXR have been found in the LBD region, including G443A (R148Q), C472A (Q158K), A488G (D163G), G1108A (A370T), T1135G (C379G), C1141T (R381W), A1207G (I403V), T1258A (F420Y), and C1276G (Q426E) 17. The G443A (R148Q) variant has no effect on PXR transcriptional activity 20. The C1141T (R381W) and A1207G (I403V) variants also have shown varying reduction in PXR-mediated transactivation 20. In COS-1 cells, *in vitro* transient transfection with the Q158K, D163G, and R381W variants of PXR, demonstrates that these three variants lead to PXR nuclear translocation as well as the reduced ligand-dependent transactivation. D163G makes PXR completely lose transactivation activity in LS174 T cells, reduce CYP3A4 induction by corticosterone, whereas enhances promoter-dependent induction by RIF 21. The rs1054190-TT genotype, which locates at the 3'UTR of PXR, reduces mRNA expression of PXR, and can be used as a marker of poor prognosis 22. Currently, PXR SNPs (rs3732359, rs3732360 and rs3814058) have been linked to the decreased percentage in nadir haemoglobin, suggesting increased docetaxel-associated toxicity in nasopharyngeal carcinoma patients and PXR (rs3814058C>T) polymorphism increases the risk of lung cancer in smokers 23, 24. We have summarized these important SNPs and their functions in** Figure 2A**. Due to the specificity of PXR SNPs, further research is needed for the precision clinical medicine.

Most studies focus on PXR's role as a transcriptional factor, whereas emerging evidences suggest that PXR can regulate gene expression at translational levels. Recently, the protein-protein interactions centered by PXR have raised much attention, especially the interactions between PXR and histone deacetylate transferase, histone arginine methyltransferase, kinase and phosphatase, and ubiquitination enzyme, indicating that PXR is implicated in posttranslational modifications (e.g., acetylation, phosphorylation, and SUMOylation, ubiquitination) and ultimately affects its transcriptional regulation and metabolic detoxification process. Protein-protein interactions also have provided a novel insight that PXR protects against toxic insult and plays important roles in the prevention of carcinogenesis. A thorough understanding of the regulation and function of PXR in carcinogenesis will help improve the diagnosis and treatment of cancer in the clinic.

Additionally, due to modulation of PXR on metabolic detoxification, inflammation, and DNA damage repair, the cell tolerance to genotoxic agents is increased, therefore, PXR also contributes to chemotherapeutic drug resistance. So far, numerous PXR antagonists have been invented and applied in clinical cancer therapy with caution to overcome drug resistance.

In this review, we have summarized the post-translational modifications of PXR, and have put much emphasis on the protective mechanisms of PXR against carcinogenesis. Additionally, we have discussed about the current understanding of PXR-induced drug resistance mechanisms to chemotherapy. We will focus on the new shreds of evidence about interaction protein network centered by PXR, including some progresses on the PXR involved DNA damage signaling.

## The structure of the PXR protein

The full-length PXR contains 434 amino acids 25, 26. The DNA binding domain (DBD) is more conserved between humans and other species. There is a bidirectional nuclear localization sequence (NLS) and two zinc finger structures: CX2CX13CX2C (zinc finger I) and CX5CX9CX2C (zinc finger II) in the DBD region, which is quite similar as that of peroxisome proliferator-activated receptor γ (PPARγ) 27 **(Figure 2B)**. Each zinc finger structure consists of four cysteine residuals that chelate a zinc ion 13, 27, 28. The ligand binding domain (LBD) of PXR is composed of a seven-member α-helical sandwich, assembled in three layers with an antiparallel five-strand β sheet spherical structure, which accommodates more ligands in this region 29. The amino acid sequence of LBD in human PXR is only 75%-80% homology with that of rats 30. Compared with other nuclear receptors, the LBD of PXR is wider and more flexible, which makes it easier to bind with many hydrophobic compounds 31. The LBD serves as the docking site for ligands and contains dimerization motifs and transcriptional activation function domains (AF-2) helix. The binding of a ligand to the LBD results in a conformational change in the AF-2 helix, and this change allows the nuclear receptor to interact with accessory proteins and to regulate the expression of target genes 32, 33.

## PXR undergoes epigenetic modifications through protein-protein interaction

PXR may be modified by acetylation, phosphorylation, ubiquitination, and SUMOylation through protein-protein interactions** (Figure 2C)**. In the following part, we will highlight the recent studies about the interactions between PXR and histone methyltransferase, histone acetylate transferase, and E3 ubiquitin ligase. The interaction network-centered by PXR will uncover the multifunctional property of PXR in different signaling pathways.

### The interactions between PXR and histone methyltransferase, histone acetyltransferase (HAT)/ histone deacetylate transferase (HDAC)

Protein arginin methyltransferases (PRMT1) is a major histone methyltransferase that associates with PXR. PXR agonist RIF recruits PRMT1 to the promoter region of CYP3A4, and in the meantime, methylates arginine 3 of histone H4. It has been proved that PRMT1 is indispensable for PXR-regulated CYP3A4 expression. Interestingly, the interaction between PXR and PRMT1 appears to have a mutual effect on both PRMT1 localization and PXR epigenetic modifications 34.

The acetylation status of PXR is quite important during its activation 35. The lysine (K) 109 in the hinge region on PXR is the major site to be acetylated by E1A binding protein p300, and the acetylation of K109 represses PXR transcriptional activity by reducing DNA-bound capability, further inhibits the formation of heterodimer with RXRα 36. Besides, steroid receptor co-activators (SRCs) facilitate PXR transcriptional modulation by recruiting endogenous histone acetyltransferases (HAT) to loosen tensed chromosome structure, thus lead to nucleosome remodeling and covalent modifications on histone tails, such as histones H3K4 methylation, H3K9 acetylation, H4K20 acetylation, and phosphorylation of the linker histone H1. Hence, acetylation has been seen as a promotional signal for transcriptional response 37. A recent study has demonstrated that PXR can form a complex with histone acetylate transferase Tip60 and augments the latter protein's enzyme activity, thus regulates colon cancer cell migration and invasion 38, suggesting that Tip60 might be one of co-activators of PXR 35, 39.

Normally, silencing mediator of retinoic acid and thyroid hormone receptor (SMRT) forms a repressor complex with histone de-acetylase (HDAC) 1 and nuclear receptor corepressor (NCoR), which inhibits PXR activity. Under this situation, HDAC activity creates a repressive chromatin environment 40, 41. The interaction between PXR and SMRT occurs within the LBD of PXR and the nuclear receptor (NR)-interacting domain of SMRT. Deletion of the PXR's AF-2 helix enhances SMRT binding and abolishes ligand-dependent dissociation of SMRT. Overexpression of SMRT abrogates PXR's transactivation of the CYP3A4, whereas silencing of SMRT enhances the CYP3A4 promoter reporter signal 42. PXR strongly represses vitamin D3 activation of the CYP24A1, in which PXR indirectly interacts with SMRT and prevents dissociation of SMRT from the CYP24A1 promoter 43. HDAC3-SMRT complex as co-repressor mainly regulates acetylation of ligand-dependent PXR 44. Treatment with TSA (deacetylase inhibitor) dramatically elevates acetylation level of PXR. Surprisingly, PXR agonist RIF can dampen the TSA-induced acetylation of PXR. TSA also alters the subcellular localization of PXR in primary hepatocytes which is isolated from C57BL6 mice 44. Pharmacologically, inhibition of HDAC3-SMRT co-repressor complex activity, together with TSA will lead to a synergistic transactivation of PXR.

NAD-dependent deacetylase protein Sirtuin 1 (SIRT1) belongs to the HDAC3 class, which is responsible for major deacetylation modifications on PXR protein. Research has showed that fasting-activated SIRT1 can bind with PXR and deacetylate PXR on lysine (K) 109 to promote PXR-mediated lipogenesis or lipid accumulation. Furthermore, SIRT1 opposes PXR coactivation by abolishing peroxisome proliferator-activated receptor γ coactivator 1 alpha (PGC-1a) expression, whereas PGC-1a increases PXR expression and transactivation function 35, 45.

### The interaction between PXR and Ubiquitination enzyme

So far, there are a lot of studies have reported interactions between nuclear receptors and E3 ubiquitin ligases 27, 46. Accordingly, the interaction may affect PXR transcriptional activity, which has been reviewed in ref. 46. For example, androgen receptor (AR) belongs to the nuclear receptor superfamily and its activation is critical for prostate cancer development and progression. Updated research has demonstrated that the RNA helicase DEAH-box (DHX) 15 stabilizes E3 ligase Siah2 and enhances Siah2 activity, thus ubiquitinates and degrades AR, whereas enhanced AR transcriptional activity contributes to prostate cancer progression 47. Hu et al. have reported that celastrol as an agonist of nuclear receptor Nur77, binds and promotes Nur77 translocation from the nucleus to mitochondria. In mitochondria, Nur77 interacts with tumor necrosis factor receptor-associated factor 2 (TRAF2), further stabilizes TRAF2. The stabilization of TRAF2 contributes to K63-linked Nur77 ubiquitination, inducing autophagy under inflammatory conditions 48. Another orphan nuclear receptor small heterodimer partner (SHP) is a negative regulator of bile acid and lipid metabolism in the liver. SHP physically occupies the transactivation domain of x-box-binding protein 1 (XBP1s), thereby preventing the ubiquitination and degradation of XBP1s by the E3 ligase complex cullin3-SPOP (speckle-type POZ protein), which has provided novel evidence that SHP governs ER homeostasis 49.

However, there are little evidence about the interaction between PXR and E3 ligase. One study has demonstrated that PXR interacts with ring-B-box-coiled-coil protein interacting with protein kinase C-1 (RBCK1) and ubiquitin protein ligase E3 component n-recognin 5 (UBR5), by which PXR is degraded by RBCK1 and UBR5 and finally altered xenobiotic's metabolism 50, 51. Domain mapping has revealed that the full-length of E3 ligase RBCK1 binds with PXR including UBL domain, novel zinc finger domain, and RING 1/2 domain. RBCK1 down-regulates the expression of PXR, and the down-regulation is inhibited by the application of MG132 50. Using mass spectrometry (MS) analysis and genome-wide siRNA screen, Ong. et al have reported that the E3 ligase UBR5 and tyrosine-phosphorylation-regulated kinase 2 (DYRK2) regulate PXR protein stability. PXR has been shown to be a substrate for DYRK2, and DYRK2-dependent phosphorylation of PXR facilitates itself subsequent ubiquitination by UBR5 51. Another study has demonstrated that the interaction between PXR and E3 ligase c-terminus of Hsp70-interacting protein (CHIP) exists in both the cytoplasm and nucleus. Threonine (T) 408 on PXR is phosphorylated by protein kinase C (PKC) and then recruits CHIP, to modulate cancer cell autophagy 52. PXR ubiquitination can be increased by protease inhibitor MG132, facilitated by the activation of the protein kinase A (PKA) signaling pathway 39. Interestingly, PXR can be heavily mono-ubiquitinated on K109, K160, K170, K198, and K226, which sets PXR in a silent state. The ubiquitination on K198 and K226 is abrogated with treatment with TSA whereas the formation of K48-linked ubiquitin chains at K170 strongly promotes the degradation of ligand-activated PXR 53. These evidences supports that the acetylation status of PXR affects its ubiquitination modification.

Interestingly, the DBD region of PXR is composed of two zinc-RING structures, which is similar as that in PPARγ **(Figure 2A)**, leading us to think about whether the zinc-RING pocket could grant E3 ligase property to PXR since PPARγ has been proved as an E3 ligase to degrade NF-κB 27. However, until now, none of the studies has been reported yet.

The PXR protein contains a SIM (SUMO-interacting motifs) consensus amino acid sequence. The PIASy-mediated SUMOylation of PXR inhibits ubiquitin-mediated degradation by the 26S proteasome and further leads to transcriptional repression of PXR during pathological states 44, 53. SUMOylation site of PXR has been found on K108 of PXR 39. K170 has been seen as a primary site of ubiquitination of PXR whereas SUMOylation of K108 and K128 abolishes ubiquitination on K170. Obviously, SUMOylation and ubiquitination of the PXR both determine its biologic function in response to xenobiotics 53. Surprisingly, the acetylation status of PXR enhances SUMOylation, whereas deacetylation modification of PXR by HDAC3-SMRT may inhibit its SUMOylation. Acetylated PXR interacts with HDAC3 and forced overexpression of SMRT further increases PXR-HDAC3 interactions 44.

### PXR contains numerous serine (S) / threonine (T) amino acids and can be phosphorylated and implicated in cellular stress response

Structurally, there are numerous serine (S) and threonine (T) amino acids in the PXR protein structure, which are easily to be phosphorylated, suggesting that the main function of PXR can be finely tuned by its phosphorylation. PKC can directly phosphorylate the T408 of PXR, which decreases PXR protein stability, followed by recruiting E3 ligase CHIP and chaperon HSP90 to modulate autophagic degradation 52. The activation of the PKA signaling pathway also increases the ubiquitination of PXR 39. The amino acid residues S8, S57, S208, S05, S350, and T408 are critical for the biological activity of the PXR protein. S57 and T408 mutationsabolish ligand-inducible PXR activity. Mutations in the PXR LBD domain at S305, S350, and T408 decrease the ability of PXR to form a heterodimer with RXRα. Mutation at position S208, S305, S350, and T408 alters the recruitment of co-factors. T408 mutation potentially affects the subcellular localization of the PXR. These data suggest that phosphorylation at specific amino acid residues can profoundly manipulate PXR biological activity 54. Besides, the phosphorylation modification at T135 and S221 of PXR decreases affinity with RXRα and co-activator SRC1 while augments the interaction with SMRT and NCoR 55, 56. Phosphatase PP1 interacts with PXR and phosphorylates T290, thus further affects PXR cellular localization and transcriptional regulation of downstream target CYP3A4, UGT1A1 57. Activated PXR recruits PP2Cα and interacts each other, leading to dephosphorylation of serum/glucocorticoid regulated kinase 2 (SGK2) at T193. Dephosphorylated SGK2 conversely augments PXR-mediated transactivation of gluconeogenic genes in human liver cells, thereby enhancing gluconeogenesis 58. In one of the recent studies, fasting-activated PXR interacts with vaccinia virus-related kinase 1 (VRK1) and phosphorylates S350 of PXR, further inhibits the sulfotransferase (SULT1E1) activity, modulating glucose metabolism 59. Phosphomimic mutation at T290 (T290D) makes PXR protein remain in the cytoplasm whereas phosphorylation at S350 may alter the binding with RXRα and its co-activators, attenuating transactivation of UGT1A1 by roscovitine 60.

**Figure 3:**
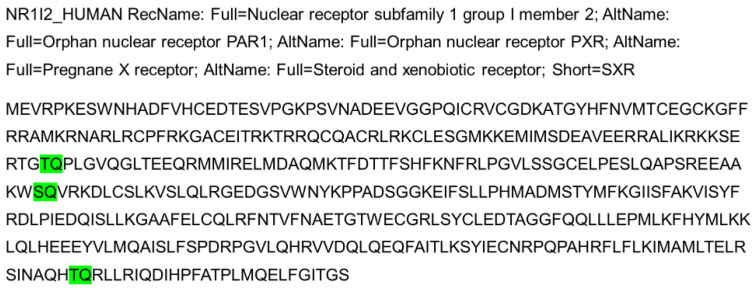
PXR protein structure contains 3 S/TQ motifs (Green color), suggesting PXR possibly is one of the substrates of ATM/ATR and is implicated in DNA damage response signaling.

Interestingly, we have also checked the protein structure of PXR, and there are three consensus S/TQ motifs, which suggest that PXR could be the substrate of ATM/ATR and phosphorylated by ATM/ATR, which may contribute to DNA damage and repair, DNA replication signaling **(Figure 3)**. The consensus S/TQ motif is the most critical structure of the substrate of ATM/ATR 61. PXR might play very important roles in DNA damage and DNA replication signaling.

## PXR as sensor and effector of xenobiotics, regulates metabolizing detoxification, glucose metabolism, and protects against carcinogenesis

As the sensor and effector of xenobiotics, PXR binds with agonists, then the corepressor complex will be released from PXR, subsequently, PXR forms a heterodimer with RXRα and then translocates to nuclear, transcriptionally regulates downstream gene's expression 62. In addition to its role in xenobiotic metabolism, PXR is implicated in regulating inflammatory responses, glucose metabolism, and protects normal cells against carcinogenesis 62-70. Most importantly, protein-protein interactions centered by PXR define specificity in signal transduction crosstalk, and influence every aspect of cellular function. In this part, we will highlight the possible mechanisms that PXR protects the normal cell against carcinogenesis.

**Figure 4:**
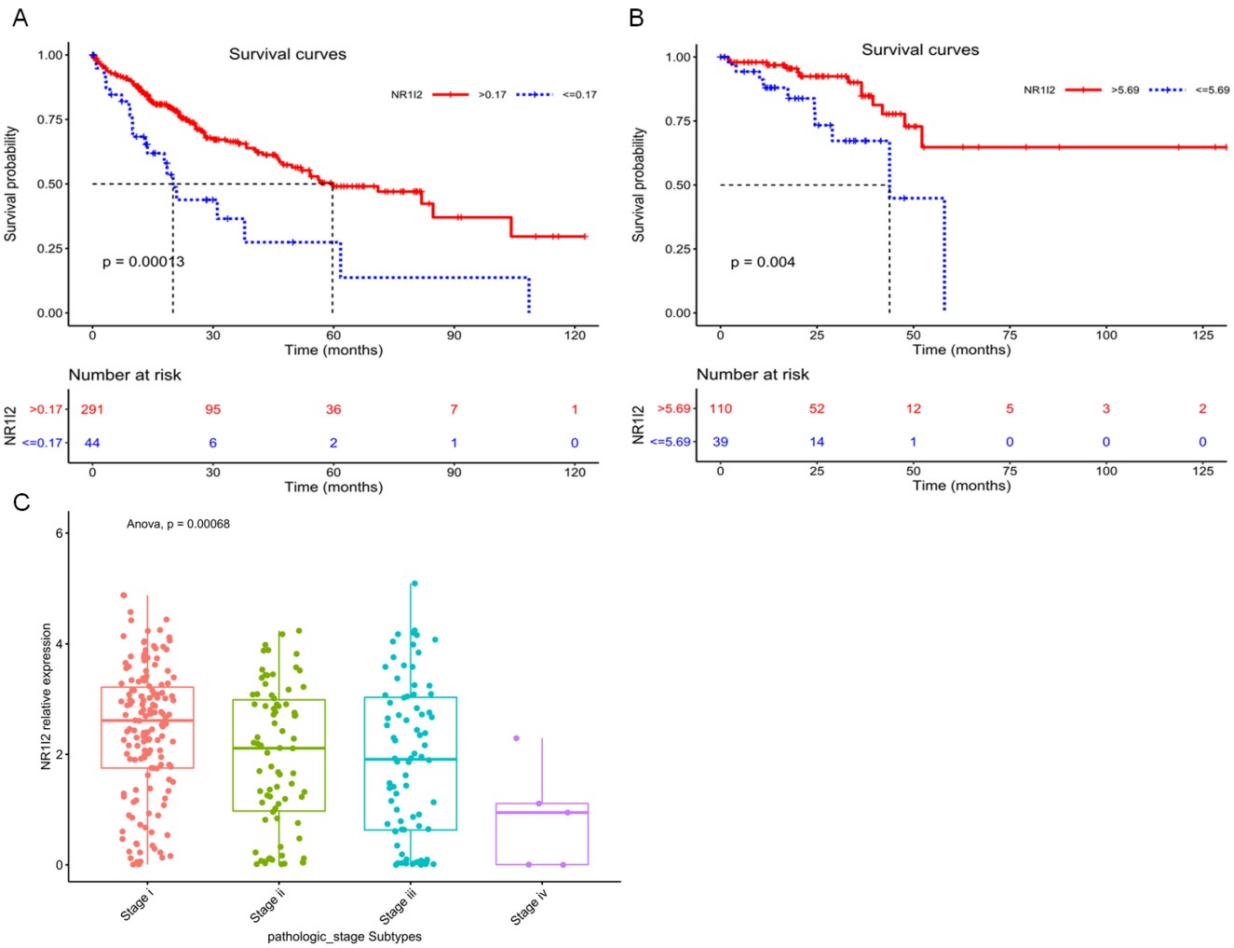
Kaplan-Meier curves for survival analysis of Liver hepatocellular carcinoma (LIHC) patients and Rectum adenocarcinoma (READ) patients based on PXR (NR1I2) mRNA expression from TCGA database. **A** Survival analysis based on the best cutoff threshold (0.17) of PXR mRNA expression in LIHC patients (335 samples) (Red line: >0.17, blue line: < = 0.17). **B** Survival analysis based on the best cutoff threshold (5.69) of PXR mRNA expression in READ patients (149 samples) (Red line: >5.69, blue line: < = 5.69). **C** PXR mRNA expression in different pathologic stages of LIHC patients (Stage I 154 samples, Stage II 78 samples, Stage III 80 samples, Stage IV 5 samples) (Anova, p = 0.00068).

Current research evidences have shown that the high expression of PXR is positively correlated with the specific survival rate of prostate cancer patients, suggesting PXR can be used as an indicator of a good prognosis for prostate cancer 71. By utilizing the cancer genome atlas (TCGA), we have analyzed the relationship between survival probability and mRNA expression level of PXR in liver hepatocellular carcinoma (LIHC) patients, as well as in rectum adenocarcinoma (READ) patients. Result shows that the high mRNA expression of the PXR is positively correlated with the survival probability of LICH and READ cancer patients **(Figure 4A and B)**, whereas negatively correlated with tumor stage and grade in LICH samples **(Figure 4C),** suggesting that PXR could be a protective factor in liver hepatocellular carcinoma and rectum adenocarcinoma.

### PXR promotes cancer cell apoptosis, inhibits proliferation, and regulates cell cycle

It has been shown that PXR is induced by PTX or cisplatin (CDDP) in a ligand-dependent manner in endometrial cancer and ovarian cancer cells 72, 73. A lot of studies have shown that PXR extensively inhibits cancer cell proliferation or induces damaged cells undergoing apoptosis to prevent carcinogenesis. In colon cancer, PXR arrests the colon cancer cell cycle at G0/G1 phase, which is mediated by upregulated p21 and downregulated E2F/RB signaling, therefore, promoting apoptosis and inhibiting the proliferation of human colon cancer cells 66. In breast cancer and cervical cancer, PXR is activated by its ligand RIF, leading to cell cycle arrest at G2/M phase and inhibiting cell growth and proliferation 74, 75. In addition, PXR also reduces Benzoapyrene (BaP) -induced genotoxicity 76, 77. However, there are also other studies have reported that activated-PXR mediates drug-induced hepatoxicity 78, 79. Therefore, PXR is a strictly context-dependent receptor, and how it plays its function depends on the post-translational modifications and cross-talk between signaling pathways 9.

### PXR activation induces metabolic detoxification enzymes to clear genotoxicity mediated by xenobiotics

So far, many CYP450 enzymes have been reported to be regulated by PXR, including Phase I metabolizing enzymes CYP2B6, CYP2C9, CYP2C19, CYP3A4, and CYP3A5; Phase II conjugating enzymes including UDP-glucuronsy transferase (UGT1A1), glutathione S-Transferase (GST), favoring detoxification of xenobiotics; some drug transporters including multidrug resistance protein 1 (MDR1/P-gp/ABCB1), multidrug resistance related protein 2 (MRP2/ABCC2) 80, 81** (Figure 1)**.

Most of the PXR-regulated drug metabolism enzymes and transporters detoxify xenobiotics and protect normal cells against carcinogenesis. Naspinski et al. have identified that PXR protects HepG2 cells from BaP-induced DNA damage by inducing CYP450 enzymes GSTA1, GSTA2, GSTM1, UGT1A6, and Breast cancer resistance protein (BCRP, ABCG2) gene expression. Additionally, the total GST enzymatic activity which favors the metabolic detoxification of BaP is found to be significantly induced in the presence of PXR 3. However, PXR-mediated GST induction is independent of the nuclear factor-erythroid 2 p45-related factor 2 (Nrf2)/Kelchlike Ech-associated protein 1 (Keap1) pathway, although Nrf2 and Keap1 have been shown to regulate GST expression positively and negatively, respectively 82. In line with Naspinski's finding, ginsenoside 20(S)-Rg3 as a functional PXR agonist significantly decreases BaP-induced DNA damage by increasing the gene expression of an important phase II detoxifying enzyme NAD(P)H: quinine oxidoreductase1 (NQO1), which is associated with the activation of Akt/Nrf2 pathway, suggesting that PXR serves as a cytoprotective factor against environmental carcinogens 77.

### PXR represses immune/inflammatory responses by inhibition of NF-κB signaling pathway

Inflammation always has been viewed as the major factor to promote carcinogenesis. PXR plays a critical role in suppressing the NF-κB-regulated signaling. Moreover, the PXR null mice display more severe inflammation in their small bowels 83, 84. Zhou et al. have shown that nuclear factor-κB (NF-κB) activation inhibits PXR/SXR and its primary target gene CYP3A by interrupting the formation of the PXR·RXRα complex, in which NF-κB p65 directly interacts with the DNA-binding domain of RXRα, thus inhibiting the transactivation regulation by the PXR·RXRα complex 2. PXR agonist pregnenolone-16α-carbonitrile (PCN) alleviates inflammatory bowel disease (IBD) symptoms, whereas mice lacking PXR expression has not been affected. Activation of PXR suppresses the expression of the NF-κB-mediated inflammatory response 85. In coordination with these findings, rifaximin, a semi-systemic rifamycin-derived antibiotic, as an intestine-specific human PXR agonist, also represses NF-κB signaling and displays a preventive and therapeutic role for the IBD model 85. Therefore, rifaximin may be a promising anticancer tool due to PXR-mediated inhibition of inflammation factors 86. Additionally, curcumin activates human PXR and significantly reduces the histological signs of colonic inflammation by induction of MDR1 expression. These data also suggest that curcumin or curcumin-like derivatives could be further developed as intestine-specific PXR activators 87. Solomonsterol A, a newly-reported PXR agonist, also prevents colitis through the reduction of NF-κB in PXR-humanized mice. These studies indicate that PXR agonists are very promising in the therapy of inflammation-driven cancer 88. Another putative mouse PXR agonist chrysin has been reported to protect against colitis by inhibiting NF-κB signal 89. It is worth to note that long-term treatment of PXR agonist to the patients inhibits the inflammatory response in the liver. Activation of the inflammatory response in hepatocytes renders modifications of SUMOylation on PXR, and SUMOylated PXR conversely further represses the immune response in hepatocytes 90.

Furthermore, many studies have reported that PXR expressed in human CD4, CD8, CD19, and CD14 cells. Upon immune stress, activated PXR inhibits the T cell proliferation, decreases the expression of CD25 and interferon γ (IFN-γ), as well as phosphorylation of NF-κb and MEK1/2 91, 92.

### PXR may promote DNA damage repair through protein-protein interaction

It has been shown that PXR protects cells against liver injury/DNA damage induced by carcinogens and anti-cancer drugs, such as polycyclic aromatic hydrocarbons (PAHs) and their metabolites, aflatoxin B 93, 94, alkylating agents (cyclophosphamide, nimustine, lomustine) 95, anthracyclines (doxorubicin, daunorubicin and adriamycin) 96 and platinum-based compounds (loplatin, oxaliplatin, cisplatin) 97. PXR interrupts the PAHS-AhR pathway and enhances CYP450 phase II enzyme activity, protecting liver cells from BaP-induced DNA damages 3, 76.

Treatment of HepG2 cells with RIF not only blocks the G0/G1 transition of the cells and inhibits cell growth, but also protects the cells from DNA damage induced by UVB 77, 98, 99. The PXR agonist ginsenoside 20(S)-RG3 activates the phosphatidylinositol 3-kinase (PI3k)/Akt/Nrf2 pathway to attenuate the DNA damage induced by BaP in human dermal fibroblasts. After inhibiting the expression of PXR by siRNA, the protective effect of ginsenoside 20(S)-RG3 has been abolished 77. The tumor suppressor gene p53 is activated when carcinogens induce DNA damage, blocking the cycle of colon cancer cells. PXR interacted with p53, and further represses its downstream genes to regulate tumor formation 100, 101.

**Figure 5:**
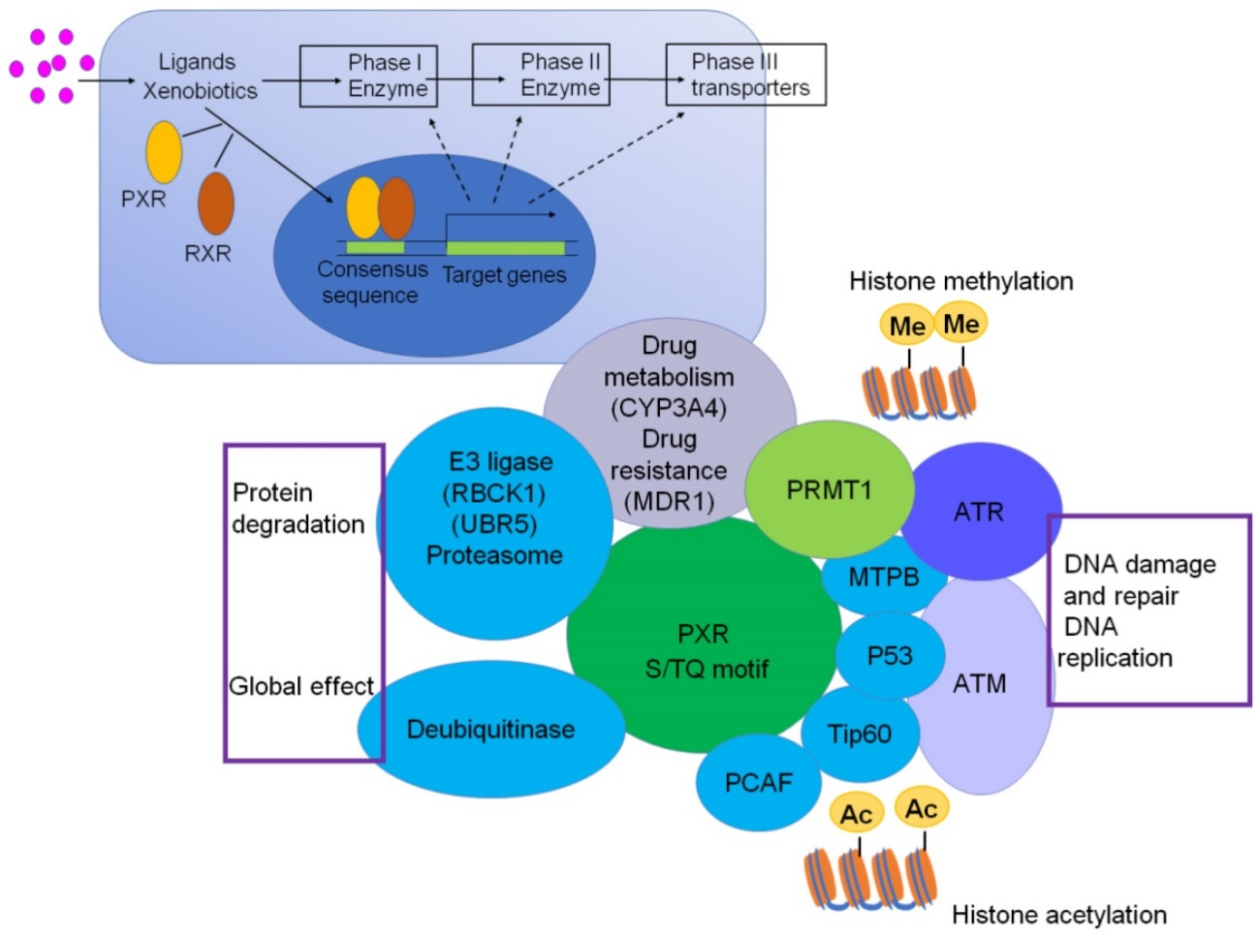
PXR centered protein-protein interactions. PXR protein structurally interacts with p53, Tip60, PCAF, MTBP, which are critical proteins to sense ATM/ATR signaling and regulate DNA damage and repair, DNA replication. PXR also interacts with PRMT1 and Tip60, thereby implicated for regulation of histone methylation and acetylation. The interaction between PXR and E3 ligase and DUB enzymes are responsible for protein degradation and ubiquitination, which might be a global effect to influence cell function. The transcriptional regulation of PXR to CYP3A4 and MDR1 makes it contribute to chemotherapeutic drug resistance.

Until now, emerging evidences have shown that PXR is implicated in protein-protein network in response to DNA damage signaling. Cui et al. have demonstrated that PXR directly interacts with AhR, and prevents the liver cancer cells from BaP-induced DNA damage through inhibiting AhR-CYP1A1 transactivation 76. Recent research has demonstrated that PXR could interact with Tip60 without ligand and augment the acetylation activity of Tip60 38. The histone acetyltransferase Tip60 closely associates with genomic instability and cancer progress. Low expression of Tip60 compromises DNA repair efficiency either under normal conditions or genotoxic stress. Further study has revealed that Tip60 regulates homology recombination (HR) to promote DNA repair 102. Additionally, Tip60 accelerates the DNA damage repair process through acetylation of ATM/ATR kinase in response to DNA damage stress 103-105. Mechanically, PXR should bear huge potential in promoting DNA damage response and repair signaling, and consequently plays protective roles in counteracting carcinogenesis, which is worth to be further investigated **(Figure 5)**.

## PXR confers anti-cancer drug resistance in the clinic

Currently, a significant barrier to effective cancer therapy is the development of drug resistance upon prolonged use. The mechanisms of drug resistance are quite complicated. The possible mechanisms include decreased drug inflow 106, increased drug efflux 106, activation of the drug detoxification system, alteration of drug targets, initiation of DNA repair reprogramming 107, regulation of cell death pathways 108, and inducing changes of microenvironments 109.

Nowadays, most chemotherapeutic regimens typically are genotoxic agents, which are designed either to damage DNA of tumor cells or to prevent the damaged DNA being repaired. Normally, DNA damage induced by genotoxic agents in normal cells can be repaired efficiently or tolerated to maintain genomic stability. Defect in DNA damage repair makes DNA lesions can't be removed timely, therefore, more and more damaged DNA accumulated and mutated, finally developed cancer. In contrast, increased repair or tolerance of DNA lesions may grant cancer cell the ability to survive in genotoxic environments upon clinical chemotherapy, thus generate resistance to DNA repair agents. DNA repair status may explain intrinsically or acquired drug resistance 110.

**Figure 6:**
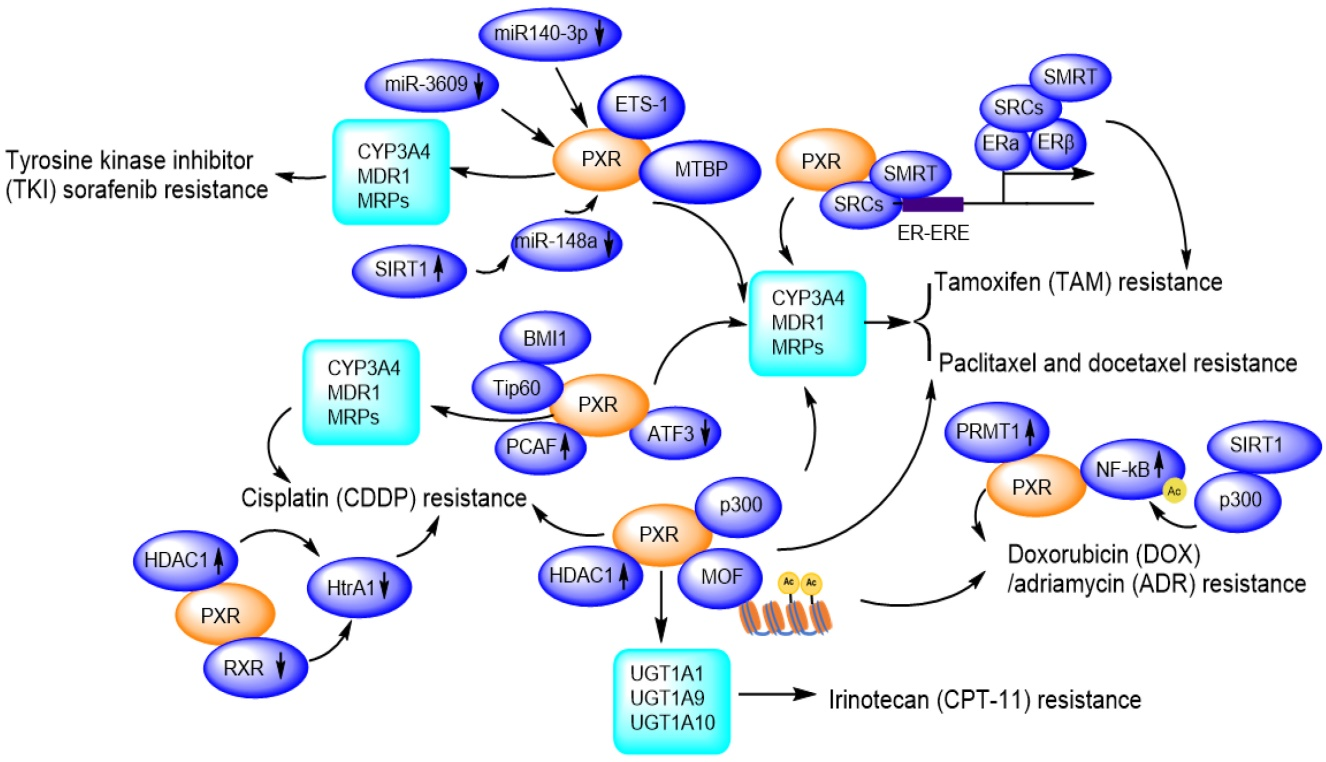
PXR confers to chemotherapeutic drug resistance including Sorafenib resistance, Paclitaxel and docetaxel resistance, Cisplatin resistance, Tamoxifen resistance, Doxorubicin/Adriamycin resistance, Irinotecan resistance through regulation of CYP450 metabolic enzymes and protein-protein interactions and transcriptional modulation. Ac: Acetylation.

Due to facilitating MDR1/CYP3A4-mediated drug metabolisms 111, and promoting DNA damage repair, PXR presents huge tolerance to genotoxic agents, thus contributes to drug resistance. So far, PXR has been reported to confer paclitaxel and docetaxel resistance 65, 112, cisplatin resistance 113, 114, tamoxifen resistance 115, 116, doxorubicin resistance 117 and sorafenib resistance 118, 119 **(Figure 6).**

### PXR contributes to paclitaxel and docetaxel resistance

In lung cancer cells A549, NCI-H358, HCC827, NCI-H1650, and NCI-H1299, upon paclitaxel treatment, the PXR mRNA and protein expression levels are elevated. The PXR expression levels are positively correlated with those of P-gp. The co-expression profile of PXR and P-gp in the peripheral blood mononuclear cells of non-small cell lung cancer (NSCLC) patients has been used as biomarker for predicting paclitaxel resistance 120. The PXR agonist SR12813 significantly increases the resistance of MDA-MB-231 cells to docetaxel, suggesting that PXR is involved in the docetaxel resistance of human breast cancer 65.

Previously, literature has reported that PXR compromises p53 function upon DNA damage response pathway 101. Coordinately, p53 is inactivated in most drug-resistant cell lines. Studies have been reported that in contrast to parental cells, p53 functionally inactive in all PTX-resistant sublines which results from two distinct point mutations in codons 236 and 239 of the DNA binding domain. This observation suggests that p53-loss-of-function may facilitate the development of paclitaxel resistance and restoring p53 tumor suppressor function, might be a promising approach to reverse chemoresistance 121, 122.

### PXR contributes to tyrosine kinase inhibitor sorafenib resistance

Hepatocellular carcinoma (HCC) is a highly heterogeneous tumor, and small molecule tyrosine kinase inhibitors (TKIs) including erlotinib, gefitinib, nilotinib, sorafenib, vandetanib, have shown dramatic therapeutic effects in cancer therapy, however, the drug resistance is an important obstacle to the successful use in the clinic. These TKIs are able to induce the expression of P-gp, which is mediated by PXR, conferring acquired TKI resistance in cancer cells 123. Sorafenib binds to and activates PXR, and promotes the expression of CYP3A4 and MDR1, facilitating the clearance or elimination of sorafenib, then HCC cells gain sorafenib-resistance to promote proliferation and invasion/migration 119. LINE-1 ORF-1p (LINE-1 ORF-1p is encoded by the human pro-oncogene LINE-1) interacts with PXR and enhances its cytoplasmic/nuclear translocation, and further mediates the resistance to and clearance of sorafenib in HCC cells by inducing CYP3A4 and MDR1 118 . Transcription factor E26 transformation specific sequence 1 (ETS-1) also binds with PXR and promotes PXR transactivation, thereby confers the sorafenib resistance in HCC tumor models. ETS-1 blockade enhances the anti-tumor capacity of sorafenib by withdrawing PXR activation 124. Conversely, miR-140-3p and miR-3609 inhibit PXR/CYP3A4/MDR1 signal, and finally result in the decelerating clearance of sorafenib in HCC cells 125, 126. Rhamnetin as an inhibitor of SIRT1, enhances miR-148a transcription thus inhibits PXR/CYP3A4/MDR1 signal, slowing down the metabolic detoxification of sorafenib in HCC cells and enhancing the sensitivity of HCC cells to sorafenib 127.

In addition to the regulation of PXR/CYP3A4/MDR1 pathway, sorafenib alone can activate p53 in a concentration-dependent manner 128. Additionally, most of HCC patients who clinically acquired resistant to sorafenib also accompany with increased P-gp expression, enhanced glycolytic metabolism, and increased nuclear factor kappa B (NF-κB) activity 129. Literature has also reported that the MDM2 binding protein (MTBP) interacts with the PXR and accumulates PXR in the nucleus, thereby facilitating PXR-CYP3A4 transactivation, which leads to the resistance of HCC cells to sorafenib. In this way, MTBP could work synergistically with PXR and might be a promising target to reverse sorafenib resistance of HCC cells 130.

### PXR contributes to cisplatin resistance

Cisplatin is a platinum compound applied for killing cancer cells and patients easily developed cisplatin resistance in clinic. In Skov-3 and OVCAR-8 ovarian cancer cells, cisplatin treatment strongly activates PXR-mediated transcription through binding with the MDR1/CYP3A4 responsive element 72, 131. Downregulation of PXR suppresses the augmented MDR1/CYP3A4 expression and significantly inhibits cell growth and apoptosis in the presence of cisplatin 72. PXR antagonist ketoconazole induces higher caspase-3 activity, whereas phenethyl isothiocyanate (PEITC) accumulates intracellular platinum level by suppressing ABCC2 protein expression, therefore, enhances the antitumor activity of cisplatin 113. Another PXR antagonist leflunomide significantly enhances caspase-3 activation in cisplatin-resistant HepG2 cells 113, 114.

A HAT gene, p300/CBP-associated factor (PCAF), as co-activator of PXR, overexpressed in cisplatin-resistant cells and endows an antiapoptotic phenotype by enhancing E2F1 expression 132. A member of the polycomb repressor complex 1 (BMI1, a proto-oncogene) recruits another HAT gene Tip60, promoting acetylation of histone H2A and H3, upregulating MDR1 expression, and contributes to acquired cross-resistance against paclitaxel, doxorubicin, as well as other chemotherapeutic drugs 133, 134.

### PXR contributes to doxorubicin (DOX) / adriamycin (ADR) resistance

Cytotoxicity experiment has revealed that the resistance of the osteosarcoma cell lines to etoposide is correlated with PXR protein expression levels and activation of CYP3A4 135. Evidences have shown that resistance to DOX can be acquired through NF-κB activation while the drug resistance to PTX can be developed through the toll-like receptor 4 (TLR4)-NF-κB pathway 117. PRMT1 as an arginine methyltransferase, interacts with PXR in MCF7/ADR cells, and this interaction could be disrupted by PRMT1 inhibitor 1 (AMI-1). AMI-1 significantly suppresses the expression of MDR1 in MCF7/ADR cells and increases cells sensitivity to adriamycin. In the term of acquired resistance, PRMT1 may be an important co-activator of PXR in regulating MDR1 gene and PRMT1 inhibitor may be promising for overcoming ADR resistance 136.

### PXR contributes to tamoxifen (TAM) resistance

PXR also is involved in the tamoxifen resistance of human breast cancer 65. An outstanding activation of PXR and persistent expression of PI3k/p-Akt has been observed, together with an elevated expression of CYP3A4 and MDR1 115, 116. Additionally, TAM-resistant MCF-7 cells (TAMR-MCF-7) express higher levels of MRP2 than parental MCF-7 cells. LY294002 (PI3k inhibitor) significantly reduces both the PXR activity and MRP2 expression in TAMR-MCF-7 cells, suggesting that PI3k plays important roles in activating PXR-MRP2 signaling and these observations might bear clinical implications during long-term TAM therapy-acquired chemoresistance 115.

### PXR contributes to irinotecan resistance

The resistance of the lung cancer cells to irinotecan (CPT-11) and its active metabolite SN38 is associated with the elevated expression of PXR downstream genes UGT1A1 and UGT1A10 137. Elevated expression of UGT1A1 and UGT1A10 enhances metabolism of SN-38, resulting in a reduced concentration of SN-38 in the lung cancer cells 138. Activation of PXR confers the resistance of colorectal cancer cells LS174T, SW480 and SW620 to irinotecan, which is mediated by the increased expression of UGT1A1, UGT1A9 and UGT1A10. The resistance can be reversed by PXR repression 138.

### The PXR genetic polymorphisms affect chemoresistance

As we mentioned above, the SNPs of PXR may alter its transcriptional regulation of CYP3A4, and MDR1, the ligand-bound capability, and cellular localization. There is no wonder that SNPs also influence pharmacokinetic profile and drug sensitivity. De Mattia et al. summarized the role of PXR polymorphisms in toxicity, efficacy, and pharmacokinetics of chemotherapeutic drugs 15. The haplotype formed by rs2276707 (39823C>T) and rs3814058 (42961T>C) linked with a lower doxorubicin clearance. An allele in rs10934498 (10051G>A) associated with decreased exposition and increased degradation of SN-38. 2654T>C polymorphism have been shown to be associated with reduced intestinal ABCB1 expression whereas the IVS617C>T polymorphism is related to intestinal CYP3A inducibility, leading to an accelerated clearance of azithromycin 19, 139. However, since SNPs vary between different people, different gender, and different study subject, the results lack independent validation. In future, new sequencing technology might provide better genetic characterizations of PXR to thoroughly understand the variability in drug sensitivity and drug resistance among individuals.

## Application and disadvantages of PXR antagonists in clinic

A lot of studies have further verified the key role of PXR antagonists in overcoming resistance.

Ketoconazole is a weak antagonist of PXR, which binds to the AF-2 of PXR instead of LBD 140. It efficiently antagonizes RIF-induced PXR activation, and inhibits the activity of CYP3A enzyme as well as MDR-1, MRP2 expression in ovarian cancer, liver cancer, and colon cancer cells. It dramatically enhances drug sensitivity such as paclitaxel and cisplatin, inhibits cell proliferation, and improves the effect of drugs 141-143. In paclitaxel-resistant SKOV-3 cells, ketoconazole significantly inhibits cell growth and proliferation, and promotes the apoptosis of drug-resistant cells 72. This effect has also been further verified in combination with cisplatin 113. Indeed, ketoconazole combination with paclitaxel or cisplatin inhibits cell growth and promotes apoptosis, increases the sensitivity of endometrial cancer cells to chemotherapeutic drugs 144. However, low-dose ketoconazole cannot directly inhibit the expression of PXR, only high-dose and long-term can inhibit the expression of PXR 16. This is consistent with the results of another study, which claimed that ketoconazole as a PXR antagonist has limitations, and cannot be applied to clinical due to its high toxicity 140. Because ketoconazole is more toxic and its clinical application is very limited, researchers are also committed to develop some of its analogs to overcome the effect of drug resistance, and find that the toxicity of FLB12 is less than that of ketoconazole, counteracting the resistance of LS174T colon cancer cells to SN-38 142.

Fucoxanthin (FUC) is a carotenoid bearing anti-inflammatory and anti-cancer effects 145. It is reported that fucoxanthin can be used as a PXR antagonist, not only promotes apoptosis of prostate cancer cells (PC-3, DU145 and LNCaP) through G0/G1 phases arrest, but also enhances the activation of caspase-3 in cisplatin-resistant HepG2 cells 114, 146. The dietary carotenoid fucoxanthin (FUC) can overcome DOX resistance in breast cancer cells (MCF-7/ADR), hepatocytes (HepG-2/ADR), ovarian cells (Skov-3/ADR) through inhibiting RIF-induced PXR, MDR1, and CYP3A4 expression at both the mRNA level and protein level. Therefore, fucoxanthin might be very promising to overcome PXR-mediated drug resistance 146.

Leflunomide as PXR antagonist is clinically used for anti-rheumatic treatment. It has an IC_50_ of 6.8μM, which can significantly enhance the activation of caspase-3 in parental and CDDP-resistant HepG2 cells 147.

Sulforaphane (SFN) is the first naturally-developed PXR antagonist *in vitro*. It is directly bound with PXR and inhibits PXR-mediated expression of CYP3A4 in human primary liver cancer cells 148. SFN arrests cancer cells in the G2/M phase, inhibits cell growth, and further inhibits the formation of DNA adducts, thus enhances the therapeutic effect of cancer 149. SFN metabolites sulforaphane-cysteine (SFN-Cys) or sulforaphane-N-acetyl-cysteine (SFN-NAC) increases the binding of phosphorylated extracellular regulated protein kinases 1/2 (ERK1/2) to α-tubulin, thus alternates the microtubule morphology and disturbs the microtubule assembly 150. Another study has reported that SFN induces apoptosis by sustained activation of ERK1/2 in NSCLC cells 151. SFN metabolites phosphorylate ERK1/2, leading to upregulation of 26S proteasome and Hsp70, as well as downregulation of βIII-tubulin, X-linked inhibitor of apoptosis protein (XIAP), Tau, Stathmin1, and α-tubulin, ultimately develops microtubule catastrophe, then reverses PTX resistance in NSCLC cells 152.

SPA70 is a newly developed antagonist of PXR by Chen's lab 153. It acts on the LBD region of PXR and blocks activation of PXR in the liver cancer cell HepG2 in a concentration-dependent manner. It suppresses the expression of multiple PXR target genes such as CYP3A4 and MDR1. SPA70 is a powerful and specific antagonist of PXR with low toxicity, which is an analogue of ketoconazole 153. Structurally, SPA70 contains a 2,5-dimethoxyphenyl component with the 5-methoxy group replacing the 5-methyl group in SJB7 (PXR agonist) 153. Nonetheless, SPA70 can work as agonist to mutant PXR (a mutation residue W299A in the ligand domain), since W299A transition is closely related to the AF-2 region of PXR contact 154. In such case, W299 amino acid is quite important for PXR activation, and the agonistic effect of CITCO also requires W299 155. Recently, the researchers have modified the four different positions of SPA70, the 4-(tert-butyl) phenyl group, the methoxy group, the hydrogen group, and the 5-methyl-1H-1,2,3-triazole ring, and have produced 81 analogs of SPA70 totally, and the binding ability of these compounds with PXR has been altered and investigated. Interestingly, although these compounds have similar structures, their functions and effects on cancer cells are quite different 156.

## Conclusions and perspectives

Since Kliewer et al. initiated PXR study in 1997, so far, it has been well studied in drug metabolisms 1. However, how PXR acts in tumorigenesis still needs further clarification. PXR *per se* is a context-dependent gene since its functional regulation occurs in multiple levels: firstly, it has high or low hypermethylation in the promoter region, which makes its gene expression silenced or overactivated 7; secondly, PXR plays its function as a heterodimer with RXRα, however, RXRα is also co-factors of many other nuclear receptors and can be affected by these nuclear receptors 26, 157; thirdly, PXR activation needs some auxiliary co-activators or co-repressors, most of which may modify PXR in post-translational modifications, thereby influence the performance of PXR 33; fourthly, the protein structure of PXR is enriched of serine and threonine amino acid, which makes PXR possibly be one of the substrates of multiple kinases and further employs with phosphorylation signaling transmission 55, 56; fifthly, the outcome of PXR binding with or without ligand varies because the process may recruit E3 ligase or co-activators or co-repressors, thus finally affects the transactivation effect of PXR 45, 52, 70. Collectively, PXR displays quite a complicated performance in response of xenobiotics. Further understanding the biological regulation of PXR will help clinical application or diagnosis.

Although PXR is a context-dependent gene, it exhibits as a protective factor in tumorigenesis, as shown by survival analysis in LICH and READ samples according to PXR mRNA expression **(Figure 4)**. PXR manipulates drug metabolism to clear the toxicity of xenobiotics, furthermore, it represses inflammation via NF-κB inhibition. The current study has resonated that PXR represses NF-κB activation whereas NF-κB activation disrupts the heterodimer of PXR-RXRα, and further inhibits PXR binding to CYP3A4 promoter 2, 83, 84. Interestingly, we found a quite similar zinc ring domain structure between PPARγ and PXR 27, which highlighted a possibility that PXR may bear similar E3 ligase activity as PPARγ to degrade NF-κB in protein level, however, this possibility needs further examined.

Although most researches focus on PXR as the transcriptional factor, PXR also regulates cross signaling through protein-protein interactions, which occurs at the protein translational or post-translational level 14. Till date, the PXR-mediated protein-protein interactions are involved in glucose and lipid metabolism, bile acid/cholesterol metabolism, inflammation regulation, ER stress, autophagy, drug metabolism, liver injury, type II diabetes, steroid/endocrine homeostasis, and carcinogenesis 62, 80. Indeed, by applying yeast two-hybrid and GST-pulldown assays, as well as an endogenous co-IP experiment, it has been found that PXR protein structurally has interactions with many proteins, which have been reviewed in ref. 33. Strikingly, PXR can interact with many critical proteins in DNA damage signaling, including p53, Tip60, PCAF, MTBP **(Figure 5)**, suggesting PXR is involved in DNA damage and repair network. There are several classical S/TQ motifs on the PXR protein structure, substantiating the fact that PXR physically might be the substrate of ATM/ATR kinase and play undefined roles in the DNA damage response pathway.

Due to the regulation of CYP3A4 and MDR1 gene expression, there is no any disputation that PXR contributes to chemoresistance in cancer therapy. PXR structurally binds to the promoter region of MDR1, MRP2 and BCRP, thus inducing anti-cancer drug resistance 158-160. Consistently, PXR activation coordinates with the expression of these drug-resistance related proteins, and inhibition of PXR by an antagonist could reverse and sensitize the response to anti-cancer drugs in patients. In the meantime, the fact that PXR promotes DNA damage response and repair provides another explanation that PXR confers to drug resistance. During carcinogenesis, PXR protects the human body against xenobiotics-induced DNA damage and maintains genome stability; however, once cancer cell gained drug resistance, the capability of promotion DNA damage repair compromises the killing effect of the anti-cancer drug to cells. At this point, PXR and tumor suppressors, such as p53, et al. work like a two-side sword in cancer therapy. Nonetheless, the mechanisms that PXR confreres multidrug resistance are not limited to transcriptional upregulation of MDR1, MRP2 and BCRP, as evidenced that SFN metabolites overcome paclitaxel resistance through microtubular disruption. The finding provokes our attention that PXR governs the receptor interaction with mitotic chromatin and the nuclear localization signal (NLS) (R66-76R) region of PXR is essential for this process 28. It is worth to note that some SNPs also influence mitotic binding of PXR. Indeed, the mechanisms that PXR-conferring multidrug resistance are quite complicated, especially its co-activators SRCs, co-repressors HDCA/SMRT/NCoR, and heterodimer partner RXRα, SNPs, governing post-translational modification-mediated drug resistance, which bears significant implications and emerged a plethora of clinical ramifications. Expanded knowledge unraveling the mechanisms of PXR-mediated drug resistance may delineate new interventive procedure for more comprehensive treatments.

Antagonist of PXR bears huge potential to overcome drug resistance. So far, many small molecules have been developed and shown potency in this field. However, the toxic effects of these drugs during administration cannot be avoided 161, 162. At present, scientists have conducted in-depth studies on the structure and metabolic pathways of PXR, using current molecular technology, computing software to develop the desired antagonist counteracting PXR-mediated chemotherapy resistance 162. Future research directions will focus on low- or non-toxic, more efficient, safer, and more specific PXR reversal agents to enhance the sensitivity of chemotherapy drugs, to explore the clinical therapeutic effects of these antagonists, and hope these inventions can solve these clinical drug resistance obstacles. Furthermore, rational drug combination therapy has yielded substantial effects in particular malignancy cases. Future evaluations are warranted in providing opportunities for the effective clinical manipulation of these PXR-mediated molecular processes.
